# Population dynamics and ecology of *Arcobacter* in sewage

**DOI:** 10.3389/fmicb.2014.00525

**Published:** 2014-11-07

**Authors:** Jenny C. Fisher, Arturo Levican, María J. Figueras, Sandra L. McLellan

**Affiliations:** ^1^School of Freshwater Sciences, University of Wisconsin-MilwaukeeMilwaukee, WI, USA; ^2^Laboratorio de Patología de Organismos Acuáticos y Biotecnología Acuícola, Facultad de Ciencias, Universidad Andrés BelloViña del Mar, Chile; ^3^Interdisciplinary Center for Aquaculture Research (INCAR)Concepción, Chile; ^4^Unit of Microbiology, Department of Basic Health Sciences, School of Medicine and Health Sciences, Institut d'Investigació Sanitaria Pere Virgili, University Rovira i VirgiliReus, Spain

**Keywords:** oligotyping, *Arcobacter*, sewage, population dynamics, V4V5, Illumina MiSeq

## Abstract

*Arcobacter* species are highly abundant in sewage where they often comprise approximately 5–11% of the bacterial community. Oligotyping of sequences amplified from the V4V5 region of the 16S rRNA gene revealed *Arcobacter* populations from different cities were similar and dominated by 1–3 members, with extremely high microdiversity in the minor members. Overall, nine subgroups within the *Arcobacter* genus accounted for >80% of the total *Arcobacter* sequences in all samples analyzed. The distribution of oligotypes varied by both sample site and temperature, with samples from the same site generally being more similar to each other than other sites. Seven oligotypes matched with 100% identity to characterized *Arcobacter* species, but the remaining 19 abundant oligotypes appear to be unknown species. Sequences representing the two most abundant oligotypes matched exactly to the reference strains for *A. cryaerophilus* group 1B (CCUG 17802) and group 1A (CCUG 17801^T^), respectively. Oligotype 1 showed generally lower relative abundance in colder samples and higher relative abundance in warmer samples; the converse was true for Oligotype 2. Ten other oligotypes had significant positive or negative correlations between temperature and proportion in samples as well. The oligotype that corresponded to *A. butzleri*, the *Arcobacter* species most commonly isolated by culturing in sewage studies, was only the eleventh most abundant oligotype. This work suggests that *Arcobacter* populations within sewer infrastructure are modulated by temperature. Furthermore, current culturing methods used for identification of *Arcobacter* fail to identify some abundant members of the community and may underestimate the presence of species with affinities for growth at lower temperatures. Understanding the ecological factors that affect the survival and growth of *Arcobacter* spp. in sewer infrastructure may better inform the risks associated with these emerging pathogens.

## Introduction

The genus *Arcobacter*, described by Vandamme et al. (1992), belongs to the family *Campylobacteraceae* within the epsilon-Proteobacteria. *Arcobacter* spp. were originally grouped within genus *Campylobacter*, but differ from campylobacters in their ability to grow under aerobic conditions and lower temperatures. The genus *Arcobacter* currently contains 18 species (Levican et al., 2013a; Sasi Jyothsna et al., 2013) isolated from diverse environments (water, plant roots, food) and hosts (humans, poultry, pigs, shellfish) (Collado and Figueras, 2011). Many *Arcobacter* species have been isolated from multiple locations, suggesting that these organisms are metabolically flexible and can survive under an array of environmental conditions.

Three *Arcobacter* species, *A. butzleri, A. cryaerophilus*, and *A. skirrowii*, have emerged in recent years as potential human pathogens (Collado and Figueras, 2011). Strains of *A. butzleri* and *A. cryaerophilus* in particular have been isolated from human stool and blood samples, and pathogenicity can range from diarrhea to bacteremia (Figueras et al., 2014). Some *A. butzleri* isolates contain a suite of virulence genes (*cadF, ciaB, cj1349, hecA, hecB, irgA, mviN, pldA*, and *tlyA*) (Douidah et al., 2012; Levican et al., 2013a) and can adhere to and invade Caco-2 cells (a gut epithelial cell line) *in vitro* (Levican et al., 2013a). The development of new DNA-based screening methods for clinical samples shows that arcobacters can often be mistaken for *Campylobacter* spp., and therefore, the potential human pathogenicity of these microbes is likely underestimated as is their role in water- and food-borne disease (Collado and Figueras, 2011; Figueras et al., 2014).

Studies of sewage and sewage-contaminated environmental waters reveal that *Arcobacter* spp. are often found in association with raw (untreated) sewage and even treated effluent water (Stampi et al., 1993; Collado et al., 2008, 2010; Cai et al., 2014). The species *A. butzleri* and *A. cryaerophilus* are the most commonly found in isolation studies, and appear to have high genetic diversity within species (Collado et al., 2008, 2010). The species *A. defluvii* and *A. cloacae* have been recently discovered in sewage samples (Collado et al., 2011; Levican et al., 2013b) as well. A culture-independent analysis of sewage using 454 pyrosequencing showed that *Arcobacter* populations accounted for approximately 4% of sewage bacterial communities, but had low diversity based on V6 pyrotag amplification (Vandewalle et al., 2012). The dominant V6 pyrotag also could not be mapped to a specific species, as this region has relatively low diversity among eight named arcobacters. So while *Arcobacter* appears to be an important component of sewage communities, relatively little is known about the diversity of these organisms or the ecological niche they may occupy in sewer infrastructure.

Here we provide an in-depth, DNA-based analysis of the *Arcobacter* community from 37 sewage samples collected in the US and Spain. The oligotyping approach sorted over 400,000 sequences into ecologically meaningful subgroups and allowed us to track changes in the *Arcobacter* populations across seasons and geography. Our findings reveal potential new species yet to be cultivated and temperature-based trends in the dominant organisms found in sewage.

## Materials and methods

### Sample collection and processing

We selected a subset of sewage samples from a larger study that contained a complete set of metadata in order to better assess the ecological factors that contribute to the distribution of total *Arcobacter* and also individual species within and among sewage samples (Tables S1, S2). All sewage samples represent a single replicate taken from municipal wastewater treatment facilities: 36 primary influent (untreated) samples collected from 12 facilities in the US on three occasions (August 2012, January 2013, and April 2013) and one sample collected from Reus, Spain in September 2012 (Table S1). These samples represent a range of geographic location, regional climate, and seasonal variation (Table S2).

Technicians at the US sewage treatment plants shipped samples on ice within 24 h of collection to our laboratory in Milwaukee, WI, USA, for processing. A volume of 25 mL of sewage was filtered (0.22 μm, 47 mm S-Pak® Millipore® filters) for each sample and filters were stored at −80°C. The sample from Reus (Spain) was a composite sample collected overnight from 8:00 p.m. to 8:00 a.m. from the inflow of the WWTP of this city. This sample was immediately taken to the laboratory at the Medical School in Reus where it was filtered. DNA was extracted following the protocol described below provided by the Milwaukee laboratory. The DNA was shipped on ice the same day to Milwaukee.

### DNA extraction, amplicon sequencing, and bioinformatic processing

We extracted DNA as previously described (Newton et al., 2013). Briefly, the FastSpin Soil DNA kit (MP Biomedicals, Santa Ana, CA) was employed according to the manufacturer's instructions using the material contained in the crushed filters. The DNA purity and concentration was assessed using the NanoDrop® spectrophotometer (Thermo Scientific, Waltham, MA) and by performing an electrophoresis in 1% TAE agarose gel.

The Josephine Bay Paul Center at the Marine Biological Laboratories in Woods Hole, MA, provided Illumina amplicon sequencing. Primers amplified the V4V5 region of the bacterial 16S rRNA gene, and the Illumina MiSeq platform produced the sequence reads. Primers, sequencing protocols, quality control measures, and bioinformatic trimming procedures for Illumina MiSeq are described in detail elsewhere (Morrison et al., 2013). The Global Alignment for Sequence Taxonomy (GAST) software (Huse et al., 2008) assigned taxonomy to our high-quality reads; this study uses only the sequences that mapped to the genus *Arcobacter*. The sequences obtained in this study are available in the National Center for Biotechnology Information (NCBI) Short Read Archive under accession number SRP047513.

### Oligotyping

GAST taxonomic classification of sequence reads (Huse et al., 2008) to the genus *Arcobacter* resulted in 408,878 sequences for oligotyping. We implemented the oligotyping pipeline (Eren et al., 2013) to determine ecologically relevant sequence groupings. Gap characters added to the ends of shorter sequences produced sequences of equal length that are required by the analysis pipeline. The “entropy-analysis” script in the oligotyping pipeline calculated the Shannon entropy at each nucleotide along the length of the sequences. The Shannon entropy provides a measure of nucleotide variation at a given position; sites that have A, G, C, and T present in approximately equal proportions among sequences have the highest entropy values, whereas highly conserved sites have a minimum entropy value near zero. Starting with the highest entropy positions along the length of the sequence, we selected 31 positions (4, 41, 54, 55, 56, 57, 58, 65, 70, 78, 85, 105, 112, 114, 115, 118, 120, 128, 130, 133, 159, 203, 212, 226, 250, 287, 301, 308, 332, 335, 343) over a read length of 375 nucleotides until entropy peaks were eliminated in individual oligotypes. We required each oligotype to have a minimum substantive abundance (−*M*, the abundance of the dominant sequence representing the oligotype) of 408 in order to reduce noise in the dataset and to focus our analysis on the more abundant oligotypes. Oligotyping with no noise filtering produced over 3800 oligotypes; elimination of oligotypes with a minimum substantive abundance of less than 408 reads (equivalent to 0.1% of the total abundance of *Arcobacter* sequence reads in the dataset) resulted in 26 oligotypes. Over 90% of sequences (372,028) were retained in the final analysis; discarded sequences were distributed evenly across samples.

### Statistical analyses

We used the vegan (Oksanen et al., 2013) and stats packages in R (R Development Core Team, 2012) for statistical analyses. Hierarchical clustering and non-metric multi-dimensional scaling (NMDS) analyses were based on Bray-Curtis dissimilarities, using oligotype matrix-count data as input. We determined the influence of environmental parameters on clusters produced by NMDS using permutation analysis of variance (ADONIS in the vegan package) with 999 permutations. The non-parametric Spearman *rho* correlation coefficients and corresponding *p* values (cor.test in the stats package) were used to determine the relationships between temperature and oligotype proportion.

### Phylogenetic analyses

ClustalW in the MEGA5 package (Tamura et al., 2011) aligned DNA sequences. The 16S rRNA sequences of *Arcobacter* reference strains represented cultivated species (Levican et al., 2013b; Sasi Jyothsna et al., 2013). We aligned the oligotype representative sequences (375 bp in length) to nearly full-length reference sequences, then trimmed to 375 bases for phylogenetic analysis. The V4V5 amplicons overlap the reference sequences from nucleotides 544–928 based on *E. coli* numbering. The Jukes-Cantor method estimated evolutionary distances, and we generated 1000 replicate trees using the Neighbor-Joining algorithm. *Campylobacter jejuni* served as an outgroup.

## Results

### Sample environmental factors and distribution of oligotypes among samples

**Figure 1:**
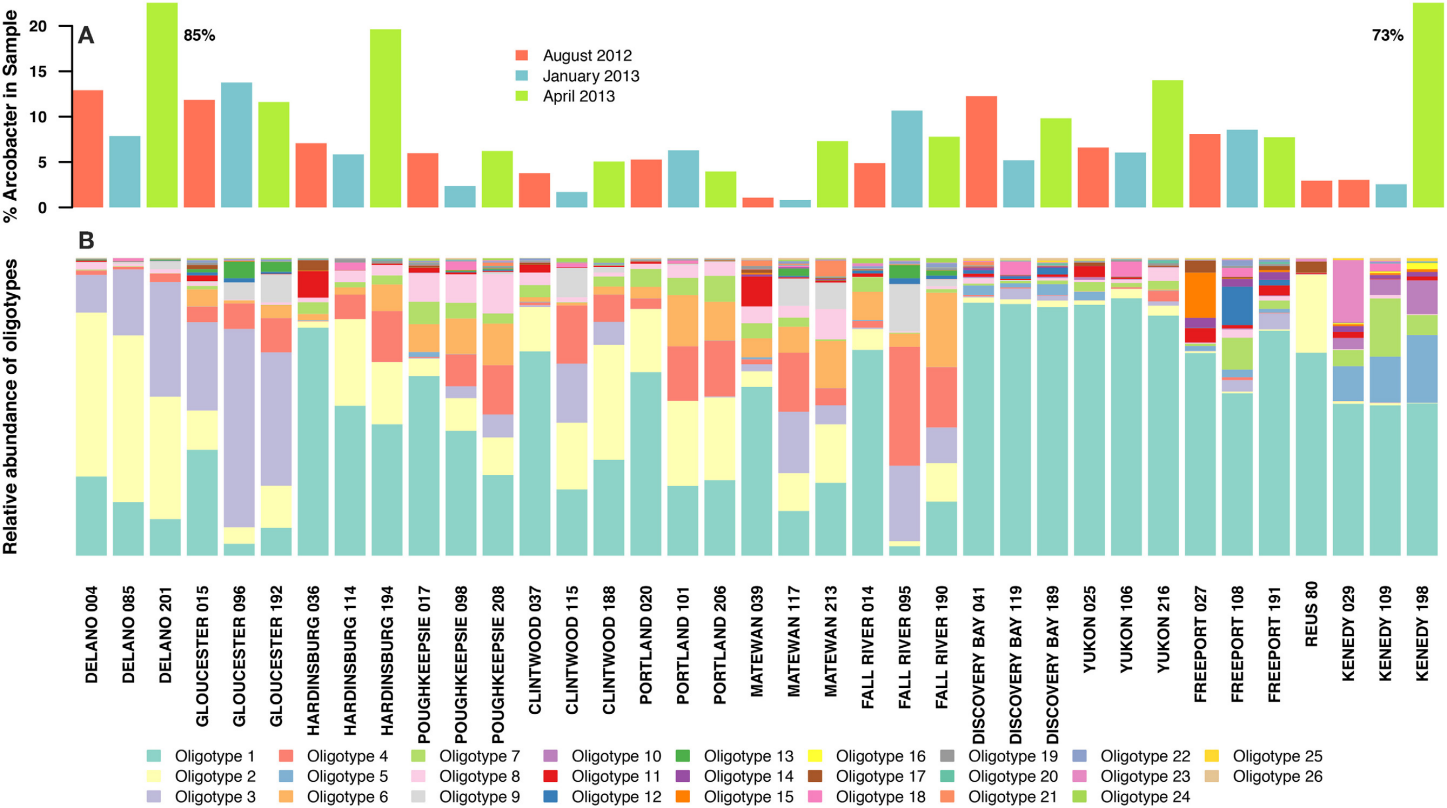
**(A)** Proportion of sequence reads in each sewage sample that mapped to the genus *Arcobacter*. Samples are color coded by the dates they were sampled: red = August 2012 (September 2012 for the Reus sample), blue = January 2013, green = April 2013. Two samples were outliers with significantly higher *Arcobacter* percentages than the rest of the samples; their values are shown as text next to the bars. **(B)** Proportions of 26 abundant oligotypes generated from sequence reads that mapped to the genus *Arcobacter* using the oligotyping pipeline. Samples are grouped by site, ordered from coldest to warmest average site temperature, then by sample collection date within sites. The legend shows the colors that represent each oligotype in **(B)**; oligotypes are numbered based on the rank of their abundance summed over the whole dataset.

The percentage of the total bacterial community that mapped to the genus *Arcobacter* ranged between 0.8 and 19.6% for most samples, and 27/37 samples had more than 5% *Arcobacter*. Two outlier samples contained 73 and 85% *Arcobacter* (Figure 1A). Over 40,000 unique sequences were present in the set of 408,878 total *Arcobacter* sequences. Alignments of the nearly complete (>1400 bp) 16S rRNA gene of *Arcobacter* reference strains (Figure S1) allowed the calculation of the Shannon entropy within the V4V5 region compared to the other variable regions; entropy within the V4V5 amplicon sequences is shown as well. The V4V5 region of *Arcobacter* reference sequences contains many high entropy nucleotide positions, although fewer than in the V2 region. Amplicons have similar high entropy nucleotide positions, but very low entropy also occurs uniformly across the length of the amplicon sequences as well. Resolution of high entropy positions produced 26 relatively abundant oligotypes (Figure 1B), while the low entropy nucleotide positions represent the high number of unique sequences that derive from microdiversity within the genus but also from sequencing noise. All 26 oligotypes in the noise-filtered analysis had a significant relative proportion (>0.9%) in at least one sample and appeared across different treatment plants and from different collection dates (Figure 1B). Oligotypes are numbered based on their total abundance rank within the dataset (i.e., Oligotype 1 had the highest overall abundance). The sewage *Arcobacter* communities from different sites were dominated by 1–3 oligotypes, with extremely high microdiversity in the minor members (represented by the noise-filtered sequences). Overall, the nine most abundant oligotypes within the *Arcobacter* genus accounted for >80% of the total *Arcobacter* sequences in all samples analyzed. The US samples, which were all untreated sewage, contained ~20 oligotypes (19.8 average, 19.5 median), but the Spain sample had only 11 oligotypes, all of which were also found in US samples. In Figure 1B, the oligotype distribution within samples is shown with samples grouped by average site temperature and by sample date within each site. Overall, oligotype distribution showed more similar patterns within sites (ADONIS *r*^2^ = 0.712, *p* < 0.001), but also appeared to have trends that corresponded to sample temperatures.

### Temperature dynamics of *Arcobacter* oligotypes

**Figure 2:**
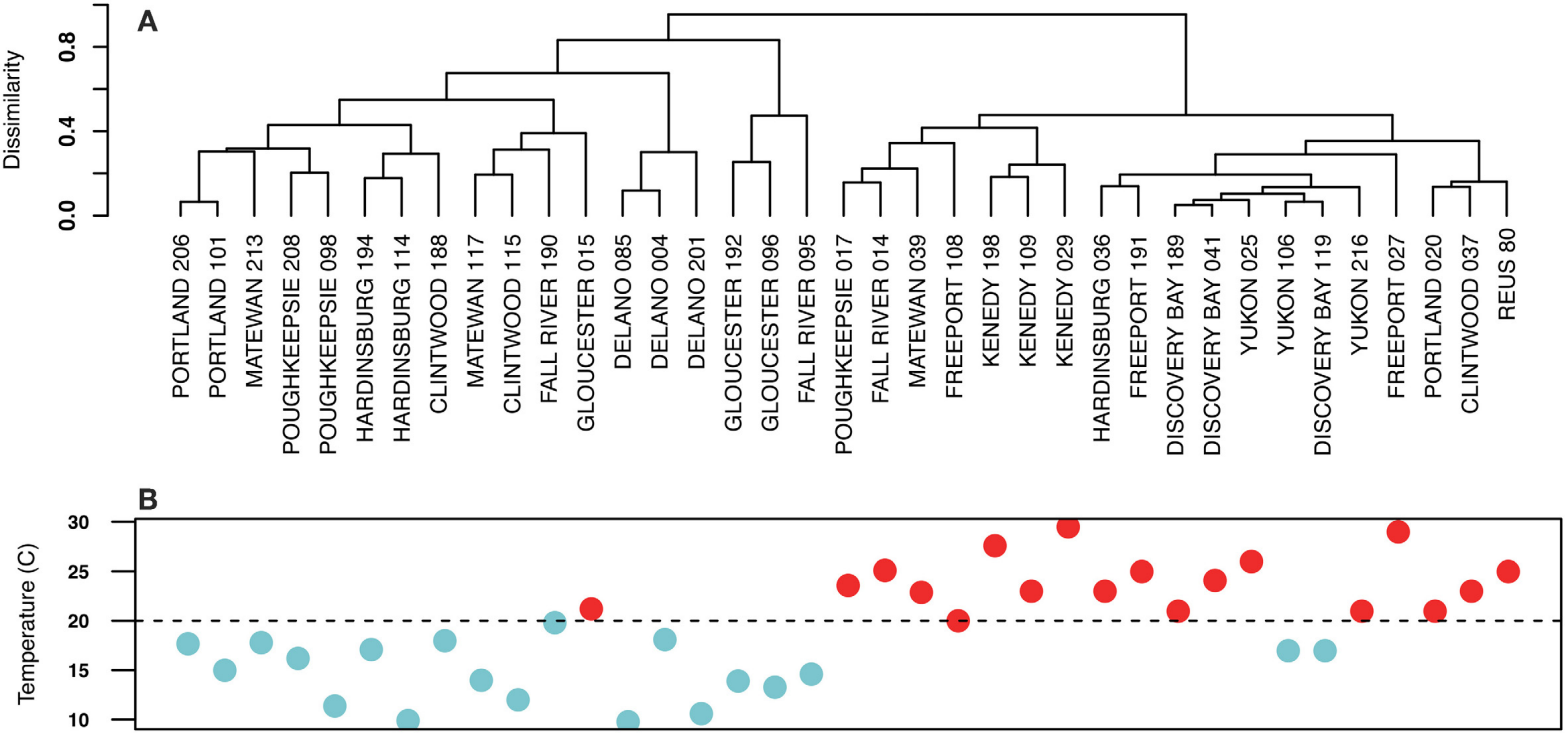
**(A)** Hierarchical clustering of sewage samples based on Bray Curtis dissimilarities of *Arcobacter* populations. Samples clustered into two distinct groups that were related to the temperature of the sample. The clade of samples on the left was composed of lower temperature samples than the clade on the right. **(B)** Temperatures of the sewage samples in **(A)** are shown below the sample name. Blue dots signify samples with temperatures below 20°C (indicated by the dashed line); red dots represent sample temperatures ≥20°C.

Figure 2A shows a hierarchical clustering analysis based on Bray Curtis dissimilarities of *Arcobacter* population compositions as described by oligotypes. *Arcobacter* populations divided on the basis of the sample temperature, with the division occurring at temperatures higher or lower than 20°C. Samples of similar temperatures taken from the same site also tended to group together. Sample temperatures in the “warm” cluster ranged from 20 to 29.5°C, with two outliers that were 17°C (Figure 2B). The “cool” cluster sample temperatures ranged from 9.8 to 19.8°C, with one outlier at 21.2°C.

**Figure 3:**
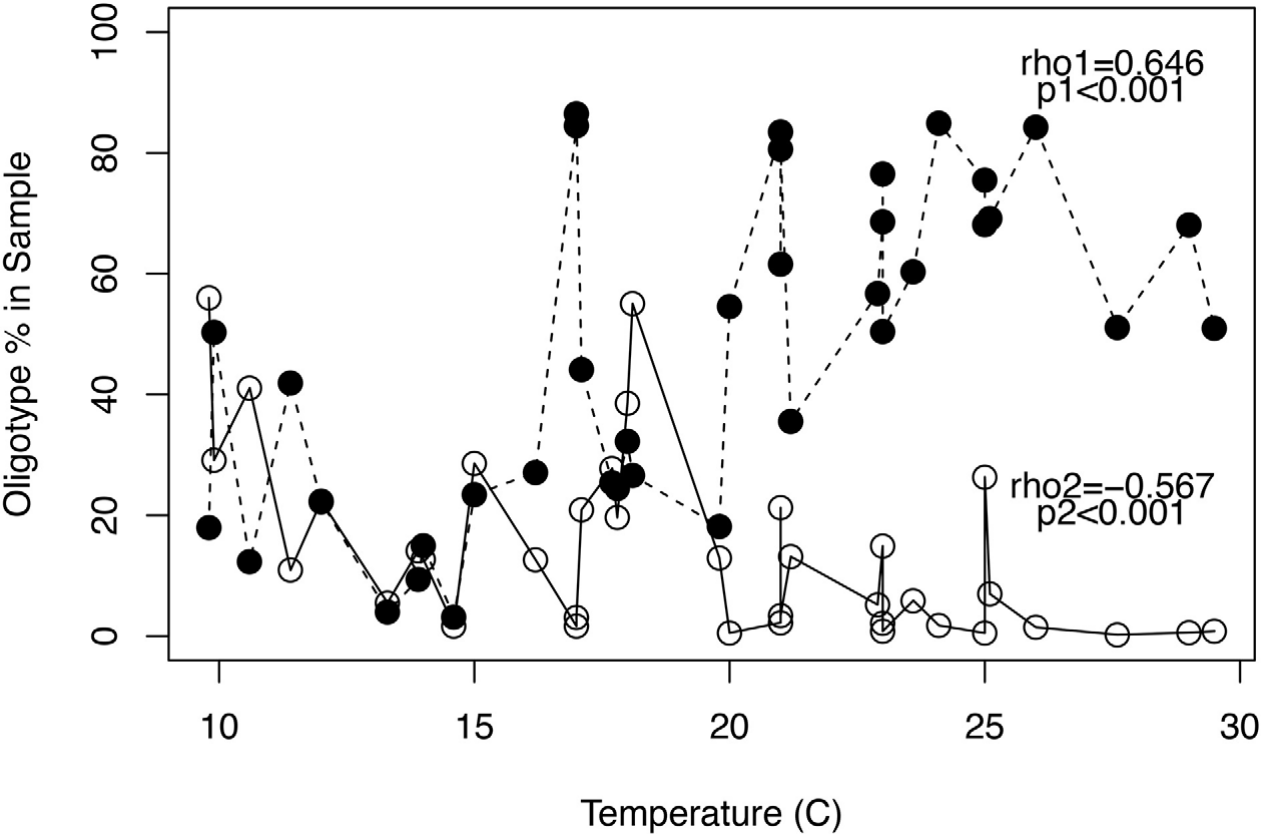
**Changes in oligotype proportions with temperature**. The two dominant oligotypes based on their relative abundance across all samples showed opposing dynamics with changes in temperature. The proportion of Oligotype (•) increased at temperatures >20°C, while Oligotype (°) proportions decreased above 20°C. The non-parametric correlation coefficient (Spearman's rho) and significance values for the relationships between oligotype proportion and temperature are shown by their respective oligotype. Temperature-proportion correlation coefficients and *p* values for all 26 oligotypes are given in Table S3.

Oligotype proportion in samples was significantly correlated to temperature for 12 of the 26 oligotypes. Eight of these tended to have a higher proportion with higher temperature (positively correlated), and 4 negatively correlated with higher temperature. The Spearman correlation coefficients and *p* values for all oligotypes are shown in Table S3. No other metadata (total suspended solids, biochemical oxygen demand, total nitrogen, total phosphorus, population size, or average daily flow) had a significant correlation to the proportion of a given oligotype present in a sample (data not shown). The two most abundant oligotypes, which are denoted as “Oligotype 1” and “Oligotype 2” displayed opposing dynamics that coincided with the temperature of a sample; i.e., they correlated positively and negatively with temperature, respectively (Figure 3). While Oligotype 1 had by far the highest proportion overall and made up over 50% of almost all the high temperature samples, its relative abundance was notably lower in most lower temperature samples. Oligotype 1 accounted for >80% of the *Arcobacter* sequences represented by oligotypes in the two 17°C samples (Discovery Bay-119 and Yukon-106) that grouped with the “warm” cluster, while the 21.2°C sample from the “cool” cluster (Gloucester-015) had <50% Oligotype 1.

### *Arcobacter* species represented by oligotypes

**Figure 4:**
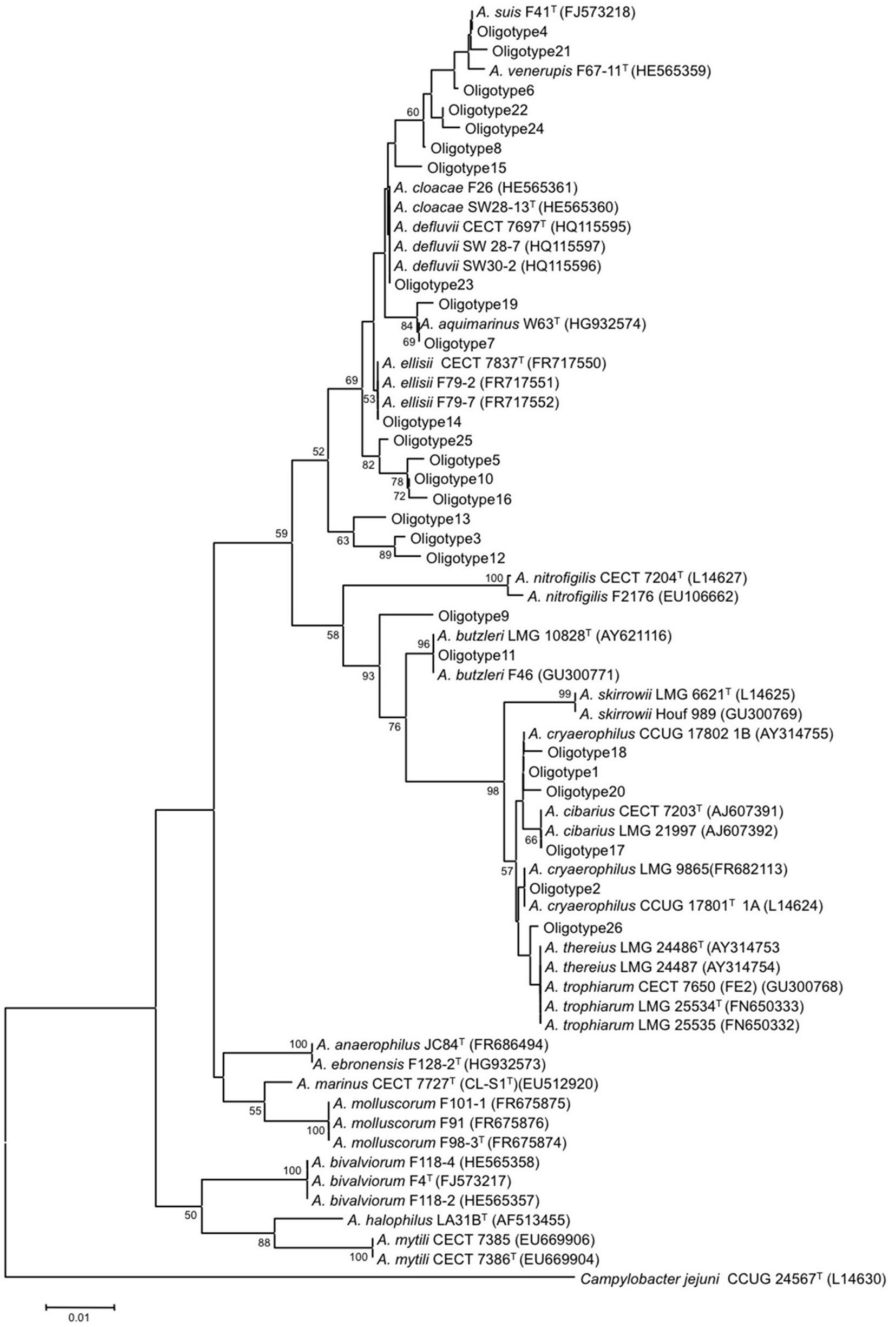
**Neighbor-joining tree of *Arcobacter* reference strains and sewage oligotypes**. Evolutionary distances were estimated using the Jukes-Cantor algorithm. This tree represents a composite of one thousand replicate trees; branches that occurred >50% of the time are noted at the nodes. The scale bar represents 1% nucleotide substitution. *Campylobacter jejuni* served as the outgroup.

Seven of the sequences representing *Arcobacter* oligotypes shared 100% identity with previously characterized *Arcobacter* species based on BLAST comparison of sequences against the NCBI nucleotide database (Table S4). Oligotype 1 and Oligotype 2 matched exactly to *A. cryaerophilus* subgroups 1B and 1A, whose 16S rRNA genes differ in the V4V5 region by only a single nucleotide. Other oligotypes with 100% identity to the type strains of *Arcobacter* species included Oligotype 4 (*A. suis*), Oligotype 11 (*A. butzleri*), and Oligotype 14 (*A. ellisii*), and Oligotype 17 (*A. cibarius*). Oligotype 23 shared 100% identity with strains of two different species (*A. cloacae* and *A. defluvii*) that were identical in V4V5 region. The majority of oligotypes had no exact matches to the type strains or other strains within cultivated species, although all were at least 98% similar to a characterized *Arcobacter* species. Figure 4 shows the phylogenetic groupings of the sequences representing the 26 oligotypes in relation to known *Arcobacter* species. Oligotype 3 formed a distinct new clade along with Oligotypes 12 and 13, and Oligotypes 5, 10, 16, and 25 also formed a clade that might represent new species. However, as the phylogenetic analysis was limited to only 375 nucleotides, the groupings of the oligotypes are more illustrative than definitive; full-length sequences would be needed to confirm the true phylogenetic relationships.

## Discussion

### Oligotyping discerns ecologically relevant patterns within the genus *Arcobacter*

In our study, different *Arcobacter* species were present in higher numbers depending on the sample temperature, similar to a previous study from estuarine water using conventional culturing methods and genetic identification of the isolates (Levican et al., 2014). For instance, Oligotype 11 (*A. butzleri*) was present in almost all samples collected during August but only in a few samples collected during January or April (Figure 1B) and correlated positively with higher environmental temperatures (Table S3). Along the same lines, Levican et al. (2014) observed a seasonal distribution of the species *A. butzleri* with a significantly higher recovery during summer. Moreover, in the later study the species *A. cryaerophilus, A. skirrowii*, and *A. nitrofigilis* were only isolated from environmental samples when water temperatures were lower (from 7.9°C to 18.2°C); however, the low number of strains recovered did not allow significant correlations to be made between species and either the water temperature or with the culturing approach (Levican et al., 2014). Conversely, the larger dataset used for oligotyping in the present study allowed us to infer a significant correlation between 12 and 26 oligotypes and the environmental temperature.

The fact that the proportions of the most abundant oligotypes varied by site and by temperature suggests that while a set group of organisms may be adapted to the ecological niche represented by a locale, changes in the environment within that system may favor different species at different times. Sewer systems appear to supply a unique niche where *Arcobacter* species thrive. Multiple samples taken from the different WWTPs demonstrate the consistency of community members at each site, but also the seasonal dynamics within populations that occur (Vandewalle et al., 2012). Dominant oligotypes were consistently present in samples collected from the same site, which may indicate a kind of ecological adaptation to general regional conditions such as climate, or more specifically, to the conditions found in particular sewerage systems.

### Abundant oligotypes represent both characterized and uncultivated species

Oligotyping is often used to compare samples from sites that have obvious ecological differences and where one might expect differentiation of populations based on environmental influences (Eren et al., 2013; Reveillaud et al., 2014). We also used oligotyping to assess the relevant nucleotide signature positions that determine speciation (Eren et al., 2013). Operational Taxonomic Unit analysis can group sequences at a fine level (>97–99% similarity), but the 16S rRNA sequences of known *Arcobacter* species do not vary by a fixed percent. By using changes in the evolutionarily relevant nucleotide positions, we were able to identify significant groupings within the *Arcobacter* genus regardless of the overall degree of sequence similarity. This analysis showed that there are a limited number of dominant ecotypes, despite the high microdiversity within *Arcobacter* sequences.

In a previous study of sewage samples using 454 pyrosequencing of the 60 bp V6 region, a single dominant sequence comprised >80% of the sewage reads that mapped to *Arcobacter* (Vandewalle et al., 2012). The reduced diversity observed in these samples could either be due to a single dominant strain (as was observed for Discovery Bay samples), or because the V6 region is highly conserved among several *Arcobacter* species. The sequence of the dominant V6 pyrotag had a 100% match to *A. cibarius, A. cloacae, A. cryaerophilus, A. defluvii, A. skirrowii, A. suis*, and *A. venerupis* (data not shown). The genetic information contained within the V4V5 amplicons in this study vs. the V6 region in the previous study allowed better resolution of these reference strains from each other, but still failed to resolve some species (e.g., *A. trophiarum* and *A. thereius*). Amplicon sequencing of the V2 region (which has the highest diversity among named *Arcobacter* species) might therefore reveal even greater *Arcobacter* diversity in sewage, but more importantly, might better resolve the most abundant ecotypes and clarify their relation to known species.

Dominant oligotypes in the sewage samples examined here share 16S rRNA gene sequence identity with named species or ecotypes as well as yet to be characterized, possible new species. Several sewage oligotypes map to arcobacters cultivated from diverse sources: e.g., *A. defluvii* and *A. cloacae* were originally isolated from sewage (Collado et al., 2011; Levican et al., 2013b); *A. venerupis, A. suis*, and *A. ellisii* are associated with food products (Figueras et al., 2011; Levican et al., 2012; Hausdorf et al., 2013); and *A. cryaerophilus* and *A. butzleri* are found in diseased animals including humans (Collado and Figueras, 2011).

At least five different groups, each containing multiple oligotypes, appear to represent new uncultivated clades. Eight of the fifteen most abundant oligotype representative sequences had no exact match, and two had closest matches to uncharacterized environmental isolates. The third most abundant had only a 98% match to the closest named species and 100% shared identity with an environmental (non-sewage) isolate. Public sequence databases such as NCBI also contain many 16S rRNA gene sequences of uncultivated and not-yet-described *Arcobacter* species, some of which come from activated sludge and sewage (Collado and Figueras, 2011). Our results demonstrate that oligotyping can be used as a highly reproducible alternative to other sequence grouping methods to elucidate the population diversity of species within a given genus and may even enable recognition of new species.

The two most abundant oligotypes in this study, Oligotype 1 and Oligotype 2, corresponded with 100% match to two different subgroups of the species *A. cryaerophilus* (subgroups 1B and 1A, respectively). Isolates from subgroup 1B are the second most commonly isolated *Arcobacter* species after *A. butzleri* using traditional culturing and molecular identification methods, but subgroup 1A is rarely recovered in this manner (Collado and Figueras, 2011). Conversely, *A. butzleri*, the predominant species typically recovered from sewage by culturing in other studies (González et al., 2007; Collado et al., 2008, 2010) was only represented by the eleventh most abundant oligotype. The predominance of *A. butzleri* by culturing could be due to the fact that most of studies include an enrichment step that favors the growth of this species; however, this assertion still needs to be experimentally verified (Levican et al., 2014).

### Implications for culturing and identification of new *Arcobacter* species

Conventional media and isolation conditions (i.e., incubation temperature, atmosphere, etc.) for the recovery of *Arcobacter* are good for isolating certain species, particularly *A. butzleri* (Collado et al., 2010). However, many uncultured strains appear to be present in sewage as well that are missed by currently used methods (Collado and Figueras, 2011). Regarding the prevalence of *A. cryaerophilus* in different studies, subgroup 1B is much more prevalent than 1A, while both groups have so far been isolated simultaneously only from food products and from animal and human clinical samples (Collado and Figueras, 2011 and references therein). The *A. cryaerophilus* strains isolated from sewage thus far have all belonged to subgroup 1B. It is not clear whether this higher prevalence of subgroup 1B is a consequence of the isolation methods used, or due to specific adaptations of these species to different ecologic niches as observed in the present study.

Different culturing methods and incubation conditions can impact the prevalence and diversity of *Arcobacter* spp. recovered from different sources (food, water, sewage, blood) (Houf et al., 2002; Levican et al., 2014). In fact, the use of direct culturing in parallel to post enrichment cultivation allowed the discovery of the species *A. defluvii* and *A. cloacae* from sewage samples (Collado et al., 2011; Levican et al., 2013b). As previously noted, the large numbers of unclassified sequences indicate that many more potential new species reside in sewage that have yet to be isolated (Collado and Figueras, 2011). Knowing which species or ecotypes grow best at different temperatures may assist in cultivating underrepresented members of the sewage community. Future studies examining both the genetic potential (through genome sequencing) and phenotypic behavior of isolates will help to better determine how these organisms grow and thrive in the sewer systems and how they may impact human health. We lack a full understanding of how the *Arcobacter* spp. in sewage relate to the arcobacters known to be pathogenic to humans and animals. Comparison of sewage oligotypes to the 16S rRNA sequences of clinical isolates may be a first step in this direction, as identification of clinical isolates by sequencing becomes more routine practice (Prouzet-Mauléon et al., 2006; Collado and Figueras, 2011).

### Oligotyping tracks *Arcobacter* population dynamics

Almost all sewage samples grouped by hierarchical clustering based on *Arcobacter* community similarity separated at a breakpoint of 20°C, which (perhaps not coincidentally) is the temperature that delineates mesophilic bacteria from psychrophilic bacteria (Willey et al., 2008). Only three samples (Gloucester-August 2012, Yukon-January 2013, and Discovery Bay-January 2013) deviated from the group prescribed by their sample temperatures. In many of the sewage samples, the top two most abundant oligotypes had opposite temperature dynamics. Additionally, Oligotype 3, which may represent a new *Arcobacter* species and Oligotype 4 (corresponding to *A. suis*) were nearly absent from the warmest sites. Similar trends in seasonal/temperature-based variation for *Acinetobacter* populations were observed in sewage samples from Milwaukee, WI. The two most abundant *Acinetobacter* V6 pyrotags oscillated in abundance over the course of the year (Vandewalle et al., 2012), and relative proportions of other genera associated with sewage infrastructure (as opposed to the fecal component of sewage) also varied seasonally (Vandewalle et al., 2012).

It is difficult to ascertain how much variation in oligotype distribution is based strictly on sample temperature, as many other factors can contribute to population dynamics. However, observed trends based on both the temperature of the sample at the time of collection and the average site temperature (approximated as the mean of collected sample temperatures) suggest that temperature may contribute significantly to determining, at the very least, the relative proportions of *Arcobacter* species present (D'Sa and Harrison, 2005; Levican et al., 2014). Knowledge of *Arcobacter* population dynamics may lead to a better understanding of risks associated with environmental releases of these organisms in the case of combined/sanitary sewerage overflows (Ashbolt et al., 2010) and provide guidance for better management in food preparations (Van Driessche and Houf, 2008; Kjeldgaard et al., 2009).

### Selective growth of *Arcobacter* species in sewage

Arcobacters make up <0.001% of the human gut microbial community (Gevers et al., 2012; Koskey et al., 2014) but they make up a significant portion of sewage samples collected from geographically diverse locations (Shanks et al., 2013; Cai et al., 2014; Koskey et al., 2014). Since *Arcobacter* is found in the human gut, albeit in low proportions, humans may be the source of *Arcobacter* to sewerage systems; however, the sewer pipe environment appears to select for their survival and growth over more dominant gut bacteria, such as the *Lachnospiraceae* (McLellan et al., 2013). Three genera previously recognized to comprise a significant portion of sewage (*Trichococcus, Acinetobacter*, and *Aeromonas*) (Vandewalle et al., 2012) are also present, but in low relative abundance, in human feces (Gevers et al., 2012; Koskey et al., 2014). Thus, the specific ecological conditions present in sewage infrastructure, generally speaking, provide an ideal niche for certain organisms, not only to thrive, but also to maintain diversity within their own populations.

The factors contributing to the survival and growth of different arcobacters are of interest because some species have been identified as emerging human pathogens (Prouzet-Mauléon et al., 2006; Collado and Figueras, 2011; Figueras et al., 2014). Species sharing genetic similarity to pathogenic strains have also been isolated from environmental waters impacted by sewage inputs (Fong et al., 2007; Collado et al., 2010) or detected in impacted waters by molecular analyses (Collado et al., 2008; Lee et al., 2012), suggesting that at least some sewage-based *Arcobacter* species are viable after sewage releases. These organisms can survive or grow at temperatures found in sewerage systems, a range of environmental water temperatures (Levican et al., 2014), and laboratory isolation conditions, which may be ≥30°C (Stampi et al., 1993; Prouzet-Mauléon et al., 2006; Levican et al., 2014). Thus, although different *Arcobacter* species may have relatively high or low optimum growth temperatures, many seem to have a wide range of survival temperatures (D'Sa and Harrison, 2005; Van Driessche and Houf, 2008). If pathogenic *Arcobacter* strains also possess this trait, they may pose a threat even at very low abundance. These findings further stress the need to better understand the genetic crossovers between human and animal pathogenic strains, sewage ecotypes, and cultured isolates in order to ascertain risks associated with *Arcobacter* species.

The results of our study can be used as a general strategy for interpreting sequence data from populations of environmental bacteria, as oligotyping provides high resolution among species, even without full-length sequences. Here we show an approach that allows differentiation of known and unknown species, and also provides information on how environmental factors can modulate the presence and relative abundance of different ecotypes. Oligotyping may provide the means to establish the links between *Arcobacter* communities from food production, water sources, sewage, and diseased humans and animals, in order to better discern patterns of survival, growth, and infection.

### Conflict of interest statement

The authors declare that the research was conducted in the absence of any commercial or financial relationships that could be construed as a potential conflict of interest.
