# Supplementary material for: Population dynamics and ecology of Arcobacter in sewage
Source: Front Microbiol. 2014 Nov 7;5:525. doi: 10.3389/fmicb.2014.00525 (PMC4224126; doi:10.3389/fmicb.2014.00525)
Supplement: Supplementary file 1 [file DataSheet1.ZIP › Supplementary_Table_S2_metadata.docx]

**Supplementary Table S2.** Metadata for sewage samples.

| **Site ID** | **State** | **Collection Date** | **Geography** | **Collection Type** | **Sample Temperature (°C)** | **Sewer Type** |
| --- | --- | --- | --- | --- | --- | --- |
| DISCOVERY BAY 041 | CA | Aug-2012 | South | Composite | 24.1 | Separated |
| DISCOVERY BAY 119 | CA | Jan 2013 | South | Composite | 17 | Separated |
| DISCOVERY BAY 189 | CA | Apr 2013 | South | Grab | 21 | Separated |
| HARDINSBERG 036 | KY | Aug 2012 | South | Composite | 23 | Separated |
| HARDINSBURG 114 | KY | Jan 2013 | South | Composite | 9.9 | Separated |
| HARDINSBURG 194 | KY | Apr 2013 | South | Composite | 17.1 | Separated |
| FALL RIVER 014 | MA | Aug 2012 | North | Composite | 25.1 | Combined |
| FALL RIVER 095 | MA | Jan 2013 | North | Composite | 14.6 | Combined |
| FALL RIVER 190 | MA | Apr 2013 | North | Composite | 19.8 | Combined |
| GLOUCESTER 015 | MA | Aug 2012 | North | Composite | 21.2 | Combined |
| GLOUCESTER 096 | MA | Jan 2013 | North | Composite | 13.3 | Combined |
| GLOUCESTER 192 | MA | Apr 2013 | North | Composite | 13.9 | Combined |
| DELANO 004 | MN | Aug 2012 | North | Composite | 18.1 | Separated |
| DELANO 085 | MN | Jan 2013 | North | Composite | 9.8 | Separated |
| DELANO 201 | MN | Apr 2013 | North | Composite | 10.6 | Separated |
| POUGHKEEPSIE 017 | NY | Aug 2012 | North | Composite | 23.6 | Combined |
| POUGHKEEPSIE 098 | NY | Jan 2013 | North | Composite | 11.4 | Combined |
| POUGHKEEPSIE 208 | NY | Apr 2013 | North | Composite | 16.2 | Combined |
| YUKON 025 | OK | Aug 2012 | South | Grab | 26 | Separated |
| YUKON 106 | OK | Jan 2013 | South | Composite | 17 | Separated |
| YUKON 216 | OK | Apr 2013 | South | Grab | 21 | Separated |
| PORTLAND 020 | OR | Aug 2012 | North | Grab | 21 | Separated |
| PORTLAND 101 | OR | Jan 2013 | North | Composite | 15 | Separated |
| PORTLAND 206 | OR | Apr 2013 | North | Composite | 17.7 | Separated |
| FREEPORT 027 | TX | Aug 2012 | South | Grab | 29 | Separated |
| FREEPORT 108 | TX | Jan 2013 | South | Composite | 20 | Separated |
| FREEPORT 191 | TX | Apr 2013 | South | Composite | 25 | Separated |
| KENEDY 029 | TX | Aug 2012 | South | Composite | 29.5 | Separated |
| KENEDY 109 | TX | Jan 2013 | South | Grab | 23 | Separated |
| KENEDY 198 | TX | Apr 2013 | South | Grab | 27.6 | Separated |
| CLINTWOOD 037 | VA | Aug 2012 | South | Grab | 23 | Separated |
| CLINTWOOD 115 | VA | Jan 2013 | South | Grab | 12 | Separated |
| CLINTWOOD 188 | VA | Apr 2013 | South | Grab | 18 | Separated |
| MATEWAN 039 | WV | Aug 2012 | South | Composite | 22.9 | Separated |
| MATEWAN 117 | WV | Jan 2013 | South | Grab | 14 | Separated |
| MATEWAN 213 | WV | Apr 2013 | South | Grab | 17.8 | Separated |
| REUS 80 | Spain | Sep 2012 | South | Composite | 25 | Combined |
