# Supplementary material for: Population dynamics and ecology of Arcobacter in sewage
Source: Front Microbiol. 2014 Nov 7;5:525. doi: 10.3389/fmicb.2014.00525 (PMC4224126; doi:10.3389/fmicb.2014.00525)
Supplement: Supplementary file 1 [file DataSheet1.ZIP › Supplementary_Table_S3_rho_p.docx]

**Supplementry Table S3.** Non-parametric Spearman correlations coefficients and significance values for relationships between oligotypes and temperature.

|  | **rho** | **p** | **p<0.0019^§^** |
| --- | --- | --- | --- |
| Oligotype1 | 0.647 | 2.0E-05 | * |
| Oligotype2 | -0.619 | 5.8E-05 | * |
| Oligotype3 | -0.513 | 1.4E-03 | * |
| Oligotype4 | -0.684 | 4.2E-06 | * |
| Oligotype5 | 0.831 | 3.5E-10 | * |
| Oligotype6 | -0.312 | 6.4E-02 |  |
| Oligotype7 | 0.433 | 8.4E-03 |  |
| Oligotype8 | -0.222 | 1.9E-01 |  |
| Oligotype9 | -0.653 | 1.6E-05 | * |
| Oligotype10 | 0.657 | 1.3E-05 | * |
| Oligotype11 | 0.787 | 1.2E-08 | * |
| Oligotype12 | -0.123 | 4.7E-01 |  |
| Oligotype13 | -0.487 | 2.6E-03 |  |
| Oligotype14 | 0.551 | 4.9E-04 | * |
| Oligotype15 | 0.517 | 1.2E-03 | * |
| Oligotype16 | 0.334 | 4.6E-02 |  |
| Oligotype17 | 0.711 | 1.2E-06 | * |
| Oligotype18 | -0.229 | 1.8E-01 |  |
| Oligotype19 | 0.111 | 5.2E-01 |  |
| Oligotype20 | 0.252 | 1.4E-01 |  |
| Oligotype21 | -0.291 | 8.5E-02 |  |
| Oligotype22 | -0.098 | 5.7E-01 |  |
| Oligotype23 | 0.408 | 1.4E-02 |  |
| Oligotype24 | -0.354 | 3.4E-02 |  |
| Oligotype25 | 0.602 | 1.0E-04 | * |
| Oligotype26 | 0.395 | 1.7E-02 |  |

^§^Corrected alpha value, α=0.05/26
