# Supplementary material for: Population dynamics and ecology of Arcobacter in sewage
Source: Front Microbiol. 2014 Nov 7;5:525. doi: 10.3389/fmicb.2014.00525 (PMC4224126; doi:10.3389/fmicb.2014.00525)
Supplement: Supplementary file 1 [file DataSheet1.ZIP › Supplementary_Table_S4_species.docx]

**Supplementary Table S4**. Oligotype similarity to cultivated *Arcobacter* species.

|  | **Phylogenetic Group** | **Closest cultivated match** |
| --- | --- | --- |
| **Oligotype1** | *A. cryaerophilus* 1B | *A. cryaerophilus* 1B, 100% |
| **Oligotype2** | *A. cryaerophilus* 1A | *A. cryaerophilus* 1A, 100% |
| **Oligotype3** | uncultivated | *A. ellisii*,98% |
| **Oligotype4** | *A. suis* | *A. suis*, 100% |
| **Oligotype5** | uncultivated | *A. ellisii*, 99% |
| **Oligotype6** | uncultivated | *A. suis,* 99% |
| **Oligotype7** | uncultivated | *A. cibarius/A. cloacae/A. defluvii*, 99% |
| **Oligotype8** | uncultivated | *A. cibarius/A. cloacae/A. defluvii*, 99% |
| **Oligotype9** | uncultivated | *A. butzleri*, 99% |
| **Oligotype10** | uncultivated | *A. ellisii*, 99% |
| **Oligotype11** | *A. butzleri* | *A. butzleri*, 100% |
| **Oligotype12** | uncultivated | *A. ellisii*, 98% |
| **Oligotype13** | uncultivated | *A. ellisii*, 99% |
| **Oligotype14** | *A. ellisii* | *A. ellisii*,100% |
| **Oligotype15** | uncultivated | *A. cibarius/A. cloacae*, 99% |
| **Oligotype16** | uncultivated | *A. ellisii*, 99% |
| **Oligotype17** | *A. cibarius* | *A. cibarius*, 100% |
| **Oligotype18** | *A. cryaerophilus* 1B | *A. cryaerophilus*, 99% |
| **Oligotype19** | uncultivated | *A. cibarius/A. cloacae/A. defluvii*, 99% |
| **Oligotype20** | *A. cryaerophilus* 1B | *A. cryaerophilus*, 99% |
| **Oligotype21** | uncultivated | *A. suis*, 99% |
| **Oligotype22** | uncultivated | *A. suis*, 99% |
| **Oligotype23** | *A. cloacae/A. defluvii* | *A. cibarius/A. cloacae/A. defluvii*, 100% |
| **Oligotype24** | uncultivated | *A. suis*, 99% |
| **Oligotype25** | uncultivated | *A. ellisii*, 99% |
| **Oligotype26** | uncultivated | *A. cryaerophilus/A. trophiarum/A. thereius*, 99% |
