# Supplementary material for: Population dynamics and ecology of Arcobacter in sewage
Source: Front Microbiol. 2014 Nov 7;5:525. doi: 10.3389/fmicb.2014.00525 (PMC4224126; doi:10.3389/fmicb.2014.00525)
Supplement: Supplementary file 1 [file DataSheet1.ZIP › Supplementary_Table_S1_samples.docx]

**Table S1.** Sequencing datasets used for analysis, sample location, abundance of sequence reads that mapped to *Arcobacter* and total bacterial

| **Sequencing ID** | **Sample ID** | **State** | ***Arcobacter*** | **Total bacterial sequence reads** |
| --- | --- | --- | --- | --- |
|  |  |  |  |  |
|  |  |  |  |  |
| SLM NIH Bv4v5--1St 41 Discovery Bay | DISCOVERY BAY 041 | CA | 8,520 | 69,479 |
| SLM NIH Bv4v5--1St 119 Discovery Bay | DISCOVERY BAY 119 | CA | 3,230 | 62,381 |
| SLM NIH Bv4v5--1St 189 Discovery Bay | DISCOVERY BAY 189 | CA | 12,358 | 125,912 |
| SLM NIH Bv4v5--1St 36 Hardinsberg | HARDINSBURG 036 | KY | 5,665 | 80,110 |
| SLM NIH Bv4v5--1St 114 Hardinsburg | HARDINSBURG 114 | KY | 4,419 | 75,574 |
| SLM NIH Bv4v5--1St 194 Hardinsburg | HARDINSBURG 194 | KY | 23,967 | 122,148 |
| SLM NIH Bv4v5--1St 14 Fall River | FALL RIVER 014 | MA | 2,961 | 60,713 |
| SLM NIH Bv4v5--1St 95 FALLRIVER | FALL RIVER 095 | MA | 6,059 | 56,841 |
| SLM NIH Bv4v5--1St 190 Fall River | FALL RIVER 190 | MA | 10,404 | 133,663 |
| SLM NIH Bv4v5--1St 15 Gloucester | GLOUCESTER 015 | MA | 7,493 | 63,274 |
| SLM NIH Bv4v5--1St 96 GLOUCESTER | GLOUCESTER 096 | MA | 7,427 | 53,987 |
| SLM NIH Bv4v5--1St 192 Gloucester | GLOUCESTER 192 | MA | 13,318 | 114,705 |
| SLM NIH Bv4v5--1St 04 Delano | DELANO 004 | MN | 10,293 | 79,712 |
| SLM NIH Bv4v5--1St 85 DELANO | DELANO 085 | MN | 4,977 | 63,264 |
| SLM NIH Bv4v5--1St 201 Monticello Delano | DELANO 201 | MN | 125,086 | 146,333 |
| SLM NIH Bv4v5--1St 17 Poughkeepsie | POUGHKEEPSIE 017 | NY | 4,272 | 71,474 |
| SLM NIH Bv4v5--1St 98 Poughkeepsie | POUGHKEEPSIE 098 | NY | 1,242 | 53,006 |
| SLM NIH Bv4v5--1St 208 Poughkeepsie Poughkeepsie | POUGHKEEPSIE 208 | NY | 9,476 | 152,333 |
| SLM NIH Bv4v5--1St 25 Yukon | YUKON 025 | OK | 5,808 | 88,086 |
| SLM NIH Bv4v5--1St 106 Yukon | YUKON 106 | OK | 5,194 | 86,021 |
| SLM NIH Bv4v5--1St 216 Yukon | YUKON 216 | OK | 18,702 | 133,531 |
| SLM NIH Bv4v5--1St 20 Gresham | PORTLAND 020 | OR | 4,128 | 78,279 |
| SLM NIH Bv4v5--1St 101 Portland | PORTLAND 101 | OR | 3,591 | 57,140 |
| SLM NIH Bv4v5--1St 206 Portland | PORTLAND 206 | OR | 5,064 | 127,804 |
| SLM NIH Bv4v5--1St 27 Freeport | FREEPORT 027 | TX | 5,775 | 71,512 |
| SLM NIH Bv4v5--1St 108 Freeport | FREEPORT 108 | TX | 6,371 | 74,483 |
| SLM NIH Bv4v5--1St 191 Freeport | FREEPORT 191 | TX | 7,335 | 94,974 |
| SLM NIH Bv4v5--1St 29 Kenedy Texas | KENEDY 029 | TX | 2,215 | 73,215 |
| SLM NIH Bv4v5--1St 109 Kenedy | KENEDY 109 | TX | 1,678 | 65,940 |
| SLM NIH Bv4v5--1St 198 Kenedy | KENEDY 198 | TX | 60,984 | 83,459 |
| SLM NIH Bv4v5--1St 37 Clintwood | CLINTWOOD 037 | VA | 2,780 | 73,986 |
| SLM NIH Bv4v5--1St 115 Clintwood | CLINTWOOD 115 | VA | 1,338 | 78,823 |
| SLM NIH Bv4v5--1St 188 Clintwood | CLINTWOOD 188 | VA | 4,321 | 85,545 |
| SLM NIH Bv4v5--1St 39 Matewan | MATEWAN 039 | WV | 669 | 62,091 |
| SLM NIH Bv4v5--1St 117 Matewan | MATEWAN 117 | WV | 519 | 64,010 |
| SLM NIH Bv4v5--1St 213 Williamson Matewan | MATEWAN 213 | WV | 9,798 | 134,098 |
| SLM NIH Bv4v5--1St 80 Reus | REUS 080 | Spain | 1,441 | 49,202 |
