# Supplementary figures and images for: Population dynamics and ecology of Arcobacter in sewage
Source: Front Microbiol. 2014 Nov 7;5:525. doi: 10.3389/fmicb.2014.00525 (PMC4224126; doi:10.3389/fmicb.2014.00525)

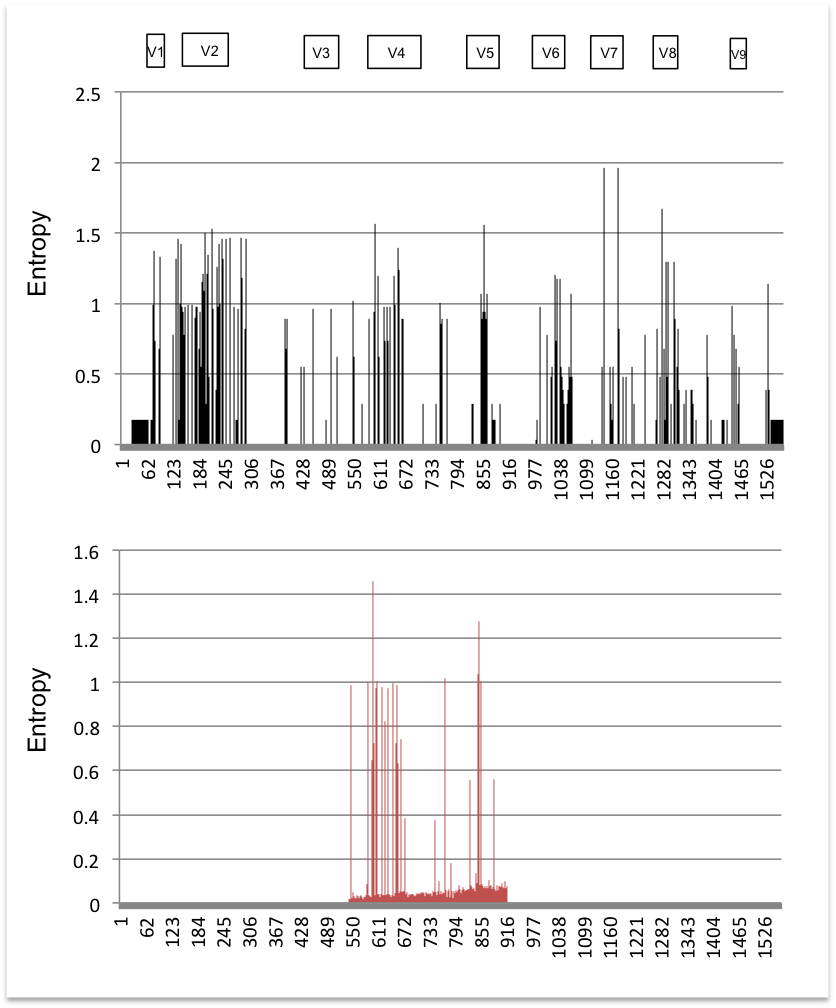

Supplement: Supplementary file 1 [file DataSheet1.ZIP › Supplementary_Figure_1_entropy.png]
